# Age and gender differences in expressive flexibility and the association with depressive symptoms in adolescents

**DOI:** 10.3389/fpsyg.2023.1185820

**Published:** 2023-04-26

**Authors:** Shaohua Zhang, Junsheng Liu, Biao Sang, Yuyang Zhao

**Affiliations:** ^1^School of Psychology and Cognitive Science, East China Normal University, Shanghai, China; ^2^Lab for Educational Big Data and Policymaking, Shanghai Academy of Educational Sciences, Shanghai, China; ^3^Department of Social Work, School of Sociology and Political Science, Shanghai University, Shanghai, China

**Keywords:** expressive flexibility, age difference, gender difference, depressive symptoms, adolescents

## Abstract

**Objective:**

This study investigated age and gender differences in the ability to flexibly enhance and suppress facial expressions according to situational demands, known as expressive flexibility (EF), as well as its relationship with depressive symptoms in adolescents.

**Methods:**

The participants included 766 Chinese high school students aged between 12 and 18 years (mean age = 14.96 years, standard deviation = 2.04; 52.2% female). Data on EF and depressive symptoms were collected using self-report questionnaires.

**Results:**

Girls scored higher on enhancement abilities than boys, but with no significant gender difference in suppression abilities. There were also no significant age-related differences in enhancement and suppression abilities. Only enhancement ability was negatively associated with depressive symptoms.

**Conclusion:**

The development of EF abilities was stable among adolescents, with varying effects in terms of gender, and the importance of EF and enhancement abilities in reducing depressive symptoms in adolescents was highlighted.

## 1. Introduction

Emotional intelligence, as a multi-dimensional construct, is defined as a set of interrelated mental processes that include appraisal, expression, and regulation of one’s own and others’ emotions, as well as the utilization of emotions in problem-solving (Salovey and Mayer, 1990). Researchers usually consider emotional intelligence as a trait or ability, namely, as trait emotional intelligence (TEI) or ability emotional intelligence (AEI). Specifically, TEI refers to a constellation of emotion-related self-perceptions and dispositions assessed by self-report questionnaires (Petrides et al., 2007), while AEI is regarded as an emotion-related cognitive ability measured *via* maximum performance tests (Mayer et al., 2008). Previous studies have reported that both TEI and AEI are associated with different physical, psychosomatic, and mental health domains or indicators in populations of various ages (Costa et al., 2021). However, despite fruitful findings on emotional intelligence and its various dimensions, expressive behaviors (especially facial expressions), as a specific emotional expression dimension have been relatively understudied and remain poorly understood. As more than half of all expressive behaviors are considered to be associated with the face (Aylett et al., 2021), there is a need to investigate the regulation of facial expressions in greater depth.

As a mirror of the internal affective state, the display of emotions has been considered to reflect emotions accurately. However, a recent meta-analysis revealed only a mild-to-moderate correlation between observation-based expressions and self-reported emotions (Durán et al., 2017). Indeed, individuals are likely to exaggerate or suppress their facial expressions to achieve particular goals in some instances. For example, individuals may exaggerate joyful expressions to impart positive impressions to others or suppress expressions of anger to avoid conflict with others. Furthermore, as emotional situations are inherently dynamic (Aldao, 2013; Aldao and Tull, 2015), the flexible use of expressive exaggeration and inhibition is crucial for individuals’ mental health outcomes (Bonanno et al., 2004; Bonanno and Burton, 2013; Aldao et al., 2014).

Expressive flexibility (EF) refers to the ability to flexibly enhance and suppress facial expressions to fit contextual demands (Bonanno et al., 2004). Bonanno et al. (2004) first developed an EF task to evaluate individuals’ ability to upregulate and downregulate their facial displays of emotions. In this task, participants are instructed to look at blocks of emotion-provoking images, then either enhance their expressions, suppress their expressions, or behave normally. Thereafter, participants’ overt emotional expressions are recorded and rated by trained observers who are unaware of the instructions and type of stimulus. Enhancement ability is then measured as the difference between the enhancement and normal conditions, while suppression ability is determined in terms of the difference between the suppression and normal conditions. In addition, EF ability has been calculated by subtracting the absolute value of the difference between enhancement and suppression scores from their sum (Westphal et al., 2010). In each case, higher scores represent better corresponding abilities.

As the previous EF task had certain disadvantages, such as low ecological validity, Burton and Bonanno (2015) developed the Flexible Regulation of Emotional Expression (FREE) scale. In this scale, adult participants are asked to indicate the extent (ranging from 1: unable to, to 6: very able to) to which they can exaggerate or inhibit their facial expressions in an array of hypothetical social scenarios. Enhancement and suppression scores are then obtained by summing the corresponding items, while the EF score is computed by subtracting the absolute difference between the enhancement and suppression scores from their sum. Higher scores indicate better flexibility in regulating emotional expressions. Several other scales have also been developed to evaluate the EF abilities of different populations, including the adolescent FREE scale for Chinese adolescents (Zhang et al., 2018), the Chinese version of the FREE scale for Chinese adults (Chen et al., 2018), and the Child and Adolescent Flexible Expressiveness (CAFE) scale for Chinese children and adolescents (Wang and Hawk, 2020). Notably, the structure and scoring formula of these scales are identical to those of the FREE scale.

Some studies have suggested that EF and/or its components (enhancement and suppression abilities) develop stably in adulthood. For example, cross-sectional research has shown that younger and older adults are equally capable of following instructions to exaggerate and inhibit facial expressions in response to emotion-eliciting images (Emery and Hess, 2011), film clips (Kunzmann et al., 2005), and musical excerpts (Sandrine et al., 2015).

However, to date, only three studies have investigated the effects of age on EF and its two components during childhood and adolescence, and the conclusions have been inconsistent. In a study by Wang and Hawk (2020), Chinese elementary and junior middle school students were asked to complete the CAFE scale to assess their self-reported EF abilities. The results indicated that, compared to their counterparts in junior middle school, participants in elementary school had significantly higher scores in all aspects (enhancement, suppression, and overall EF). In contrast, Wang and Hawk (2019) had also employed the EF task to evaluate observed EF abilities among Chinese elementary and junior middle school students across two waves at 6-month intervals. The results revealed a significant increase in the participants’ expressive enhancement, suppression, and flexibility abilities from Wave 1 to Wave 2. However, there were no significant differences between the elementary and junior middle school participants in any of the three scores. More importantly, Wang and Hawk (2019, 2020) argued that these inconsistent results may reflect the curvilinear growth trajectories of EF abilities during adolescence. In addition, Haag et al. (2022) developed the Flexible Regulation of Emotional Expression Scale for Youth (FREE-Y) to assess expressive enhancement, suppression, and flexibility in American adolescents and revealed no significant age differences in any of the three scores.

It is important to point out that, while contradictory results from the above studies may reflect nonlinear growth trajectories of EF abilities during adolescence, this interpretation was not adequately tested (especially in Chinese adolescents) due to the relatively narrow age range of the adolescent participants (aged 9–15 years old) in the studies of Wang and Hawk (2019, 2020). Therefore, the effects of age differences on EF abilities during adolescence remain unknown and require further investigation.

Three studies have also examined sex differences in EF abilities (Wang and Hawk, 2019, 2020; Haag et al., 2022), with inconclusive results. Specifically, Wang and Hawk (2019, 2020) collected cross-sectional and longitudinal data on the effects of gender on EF abilities among Chinese youth. The findings revealed that girls scored higher than boys in expressive suppression ability but not in expressive enhancement or flexibility abilities. In contrast, Haag et al. (2022) assessed EF abilities in American youth and found that girls had higher scores than boys in expressive enhancement ability but not in expressive suppression or flexibility abilities. These major inconsistencies in study findings highlight the need for further research.

Existing evidence suggests that the ability to both enhance and suppress outward expressions is associated with positive interpersonal outcomes (e.g., better friend-rated adjustment, life satisfaction, health-related quality of life, and peer acceptance). It is also associated with negative intrapersonal outcomes (e.g., lower friendship quality, distress, post-traumatic stress disorder, and anxiety; Bonanno et al., 2004; Westphal et al., 2010; Rodin et al., 2016; Strickland and Skolnick, 2020; Wang et al., 2020; Lenzo et al., 2021; Sardella et al., 2021; Shangguan et al., 2022; Wang et al., 2022). Furthermore, deficits in this ability in adults are associated with social anxiety (Maccallum et al., 2021), complicated grief (Gupta and Bonanno, 2011), and in patients diagnosed with Alzheimer’s disease (Henry et al., 2009) or schizophrenia (Henry et al., 2007).

Several studies have specifically investigated the association between EF abilities and depressive symptoms in adults. A pioneering study revealed that EF and suppression abilities were significantly associated with fewer depressive symptoms, while enhancement ability was not (Burton and Bonanno, 2015). Chen et al. (2018) replicated these findings with Chinese adults. In contrast, Rodin et al. (2016) reported that a higher observed enhancement ability, but not suppression ability, was significantly associated with decreased severity of depression and PTSD in combat veterans. A follow-up study demonstrated that the levels of observed EF abilities were predictive of a 1-month decrease in depressive symptoms and anxiety in college students with low context sensitivity (Southward and Cheavens, 2017). In addition, Chen and Bonanno (2021) identified predominant latent profiles of emotion regulation flexibility and found that all inflexible regulators exhibited greater depressive symptoms.

However, to the best of our knowledge, only two studies have evaluated the potential link between EF abilities and depressive symptoms in adolescents (Wang and Hawk, 2020; Haag et al., 2022). Wang and Hawk (2020) adopted the CAFE scale and the Child Depression Inventory (CDI) to measure self-rated EF abilities and depressive symptoms in elementary and junior middle school students from mainland China. The results showed that expressive enhancement, suppression, and flexibility were significantly associated with fewer depressive symptoms. Haag et al. (2022) utilized the FREE-Y and the CDI-2 to assess American adolescents’ self-reported EF abilities and depressive symptoms. The findings revealed that expressive enhancement, suppression, and flexibility were significantly negatively correlated with depression. However, it should be noted that the participants in Wang and Hawk (2020) study were older Chinese children and early-age adolescents, with no participants in middle or late adolescence, while the participants in Haag et al.’s study were American adolescents.

Despite being an essential aspect of emotional intelligence, the flexible regulation of facial expressions is a relatively neglected field of research. Specifically, existing research on EF has largely focused on the developmental characteristics (i.e., age and gender) of EF and its relationship with depressive symptoms in adults (Haag et al., 2022). However, to our knowledge, no study has directly investigated age and gender differences in EF, as well as the association between EF and depressive symptoms, in Chinese adolescents. On the one hand, compared to adulthood, adolescence is not only a period for youth to rapidly develop their emotion regulation skills (Zimmermann and Iwanski, 2014; Ahmed et al., 2015; Lennarz et al., 2018), but also a challenging stage in terms of potential emotional dysregulation problems, especially depressive symptoms (Lee et al., 2014; Schäfer et al., 2017; Young et al., 2019). On the other hand, compared to Western cultures, Chinese culture places greater emphasis on suppression rather than expression of emotions to promote relational and social harmony (Fischer et al., 2004; Chen et al., 2020), which may result in inconsistent conclusions pertaining to the developmental characteristics of EF and its link with depressive symptoms.

Based on the above considerations, the present study aimed to examine the effects of age and sex on EF as well as the association between EF and depressive symptoms, using well-validated self-report questionnaires in a large sample of Chinese adolescents aged 12–18 years. First, given that no direct research was identified on these aspects and that the findings from related studies have been contradictory, the present study had no specific hypothesis on the effect of age on enhancement, suppression, and overall EF abilities (Hypothesis 1). Second, in line with previous studies (Wang and Hawk, 2019, 2020), it was hypothesized that female participants would score higher than their male counterparts in suppression ability, but not in expressive enhancement or flexibility abilities (Hypothesis 2). Third, based on prior research (Wang and Hawk, 2020; Haag et al., 2022), it was hypothesized that EF, as well as enhancement and suppression abilities, would be significantly associated with fewer depressive symptoms in adolescents (Hypothesis 3).

## 2. Methods

### 2.1. Participants

A total of 766 adolescents (*N* = 766, 52.2% females) aged between 12–18 years [*M* = 14.96, standard deviation (SD) = 2.04] were recruited from six schools in the Sichuan, Shandong, and Shaanxi Provinces of China. All participants were Han Chinese, which is the major ethnic group in China. The participants were divided into seven age groups:12 years (*N* = 113), 13 years (*N* = 130), 14 years (*N* = 98), 15 years (*N* = 84), 16 years (*N* = 124), 17 years (*N* = 106), and 18 years (*N* = 111). The design and data collection procedures were approved by the Ethics Committee of East China Normal University (Approval Number: HR 121-2019). Informed consent was obtained from the parents or guardians of the students.

### 2.2. Measures

#### 2.2.1. Expressive flexibility

EF in participants was measured using the AFREE scale (Zhang et al., 2018). The scale has been validated in terms of evaluating self-perceived EF and its two components (enhancement and suppression) in Chinese youth. This 25-item scale asks adolescents to indicate their ability to modulate their facial expressions in a series of standardized hypothetical positive and negative contexts. The scale responses range from 1 (not at all) to 6 (very much), and it includes expressions such as “I can exaggeratedly express my compliments when friends tell me they have won a prize in a competition which I am not interested in.” Enhancement and suppression scores are computed separately by obtaining the sum of the relevant items whereas the EF score is computed by subtracting the absolute difference between the enhancement and suppression scores from their sum. Higher scores indicate better corresponding abilities. In the current study, the AFREE scale showed appropriate internal consistency (Cronbach’s *α* = 0.87) as well as suppression (Cronbach’s *α* = 0.85) and enhancement (Cronbach’s *α* = 0.83) assessment consistency.

#### 2.2.2. Depressive symptoms

Depressive symptoms in adolescents were assessed using the Chinese version of the Center for Epidemiologic Studies Scale-Depression (CES-D; Radloff, 1977; Chen et al., 2009). This 20-item scale requires participants to respond to descriptions such as “I felt that I could not shake off the blues even with the help from my family or friends” and then rate how frequently each depressive symptom occurred in the past week, ranging from 0 (rarely or none of the time) to 3 (most of the time). Thereafter, four items are reverse-scored and summed to provide a total score, with a higher total score indicating more depressive symptoms. The CES-D demonstrated good reliability in this study (Cronbach’s *α* = 0.83).

### 2.3. Statistical analyses

IBM SPSS Statistics 21.0 software (IBM Corp, 2012) was used for the data analysis. First, analysis of variance (ANOVA) and multivariate analyses of variance (MANOVAs) were conducted, with age and gender as the between-subjects variables, to investigate age and gender differences in expressive enhancement, suppression, and flexibility abilities. Second, Pearson correlations and hierarchical linear regressions were performed, with age, sex, and EF abilities as predictor variables and depressive symptoms as the predicted variables. Specifically, in Model 1, depressive symptoms were regressed on age, gender, and EF ability; in Model 2, depressive symptoms were regressed on age, gender, enhancement, and suppression ability. This approach was undertaken to evaluate the relationship between EF ability and depressive symptoms.

## 3. Results

### 3.1. Age and gender differences in EF among Chinese adolescents

An age group × gender ANOVA with EF scores revealed no significant main effect for age (*F*(6, 752) = 0.64, *p* = 0.695, *η_p_*^2^ = 0.005), a significant main effect for gender (*F*(1, 752) = 3.97, *p* = 0.047, *η_p_*^2^ = 0.005), and no significant effect for the interaction between age and gender (*F*(6, 752) = 1.03, *p* = 0.406, *η_p_*^2^ = 0.008) on EF. In addition, a *post hoc* comparison showed a marginally significant gender difference in the EF scores, (*F*(1, 752) = 3.41, *p* = 0.065, *d* = 0.13), with girls (*M* = 100.98, SD = 18.83) scoring higher than boys (*M* = 98.46, SD = 18.80).

An age group × gender MANOVA with enhancement and suppression scores revealed no significant main effect for age (Wilks’ *λ* = 0.992, *F*(12, 752) = 0.533, *p* = 0.894, *η_p_*^2^ = 0.004), a significant main effect for gender (Wilks’ *λ* = 0.984, *F*(2, 752) = 6.148, *p* = 0.002, *η_p_*^2^ = 0.016), and no significant effect for the interaction between age and gender (Wilks’ *λ* = 0.988, *F*(12, 752) = 0.758, *p* = 0.695, *η_p_*^2^ = 0.006) on EF. Additionally, univariate *post hoc* analyses showed that gender had a significant effect on enhancement scores (*F*(1, 752) = 12.163, *p* < 0.001, *η_p_*^2^ = 0.016), although its effect on suppression scores was not significant (*F*(1, 752) = 2.238, *p* = 0.135, *η_p_*^2^ = 0.003). Moreover, the *post hoc* test showed that girls (*M* = 59.17, SD = 9.23) had higher enhancement scores than boys (*M* = 56.70, SD = 9.99), *F*(1, 752) = 12.163, *p* = 0.001, *η_p_*^2^ = 0.016, *d* = 0.26.

### 3.2. Relationship between EF and depressive symptoms among Chinese adolescents

The results of the Pearson correlation analysis indicated that EF was significantly correlated with fewer depressive symptoms (*r* = −0.080, *p* = 0.027). Notably, enhancement ability (*r* = −0.123, *p* = 0.001) was significantly associated with fewer depressive symptoms, whereas suppression ability was not (*r* = −0.019, *p* = 0.604; see Table 1).

**Table 1:** Means, standard deviations and correlations for all the main variables.

	*M*	SD	Expressive flexibility	Enhancement	Suppression
Age	14.96	2.04	−0.030	−0.031	−0.018
Gender			−0.107^**^	−0.128^***^	−0.048
Expressive flexibility	109.93	15.87	—		
Enhancement	57.99	9.67	0.812^***^	—	
Suppression	51.94	9.81	0.818^***^	0.328^***^	—
Depressive symptoms	15.64	8.70	−0.080^*^	−0.123^**^	−0.019

*M*, mean; SD, standard deviation; ^*^p < 0.05, ^**^p < 0.01, ^***^p < 0.001.

Additionally, hierarchical linear regressions were conducted to ascertain the extent to which certain factors (age, sex, EF, and enhancement and suppression abilities) could predict the levels of depressive symptoms. The results showed that neither age nor gender significantly predicted the levels of depressive symptoms in any of the models. In Model 1, EF significantly predicted fewer depressive symptoms. In Model 2, enhancement ability, but not suppression ability, significantly predicted lower depressive symptoms (see Table 2).

**Table 2:** Regression analyses for the prediction of depression by expressive flexibility among adolescents.

		Depressive symptoms						
		*B*	SE	*β*	*p*-value	Adj. *R*^2^	*F*_change_	*R* ^2^_change_
Model 1						0.005	2.244	0.009
Step 1	Age	0.208	0.154	0.049	0.178			
Step 2	Gender	−0.068	0.629	−0.004	0.914			
Step 3	Expressive flexibility	−0.036	0.017	−0.079^*^	0.029			
Model 2						0.013	3.497	0.018
Step 1	Age	0.201	0.153	0.047	0.191			
Step 2	Gender	−0.247	0.631	−0.014	0.695			
Step 3	Enhancement	−0.118	0.034	−0.131^**^	0.001			
	Suppression	0.022	0.034	0.024	0.520			

Negative values indicate a decrease in depressive symptoms.

## 4. Discussion

From the perspective of emotional intelligence, the present study used a large sample of Chinese adolescents to investigate the effects of basic characteristics (i.e., age and sex) on EF and the relationship between EF and depressive symptoms. The results revealed that EF and its two components (i.e., enhancement and suppression) remained stable throughout adolescence. Moreover, contrary to the second hypothesis, female participants reported higher scores than their male counterparts in EF and enhancement abilities but not in suppression ability. Additionally, self-reported levels of EF and enhancement abilities were found to be significant predictors of fewer depressive symptoms, but not suppression ability.

### 4.1. Age differences in EF among Chinese adolescents

The current study found no significant effects for age on any aspect of self-rated EF ability (overall EF, enhancement, and suppression) among Chinese adolescents. These findings are inconsistent with those obtained from two related studies (Wang and Hawk, 2019, 2020) but are in agreement with those of other previous studies (Kunzmann et al., 2005; Westphal et al., 2010; Emery and Hess, 2011; Sandrine et al., 2015; Haag et al., 2022). Using a sample of older children and younger adolescents, Wang and Hawk (2019) reported mixed results regarding changes in EF abilities over time. They argued that these inconsistent findings may reflect a trough in the development of EF abilities during early adolescence. However, this hypothesis was not fully verified because the study participants were older children and younger adolescents, with no older (middle-to-late) adolescents involved. Through expanding the age range of participants, the present study was able to directly examine how EF abilities develop with age in both younger and older adolescents. The results showed that EF (including enhancement and suppression) abilities were stable throughout Chinese adolescence, which was consistent with conclusions drawn from a recent study conducted in American adolescents (Haag et al., 2022).

Furthermore, related studies on age-associated changes in EF abilities among adults have revealed that levels of EF and/or its two components (enhancement and suppression) remain relatively stable during adulthood (Kunzmann et al., 2005; Westphal et al., 2010; Emery and Hess, 2011; Sandrine et al., 2015). This study corroborated these findings in showing that EF abilities remain stable during adolescence. One possible explanation for this finding is that EF develops primarily earlier in childhood (Wang and Hawk, 2019), and thus exhibits a stable developmental trajectory during adolescence. Future research with younger and older children is needed to fully test this hypothesis.

More broadly, this study revealed that, as a core facet of emotional intelligence, EF has a trait-like quality, which may provide further empirical support for the trait approach to emotional intelligence and for regarding emotional intelligence as an affective characteristic of personality (Quattropani et al., 2022) or as a lower-order personality construct (Petrides et al., 2007). Considering the multi-dimensional nature of emotional intelligence, more research is needed to confirm this finding.

### 4.2. Gender differences in EF among Chinese adolescents

The results further showed that girls scored higher than boys in overall EF and enhancement abilities, but not in suppression ability. These findings differed from those reported previously (Wang and Hawk, 2019, 2020), which showed that Chinese females had better scores than males in suppression ability, but not in EF or enhancement ability. Nevertheless, the findings of this study can be explained from the perspective of social gender roles. Compared to individualistic cultures (e.g., American culture), collectivist cultures (e.g., Chinese culture) place greater emphasis on maintaining interpersonal harmony and encourage people to care more about the interpersonal consequences of emotion regulation behaviors (Matsumoto et al., 2008; Wei et al., 2013). Thus, Chinese females and males are encouraged to suppress their emotions when necessary (Deng et al., 2013), leading to similar expressive suppression levels in both genders. Moreover, given the established positive relationships between the levels and ability of expressive suppression among Chinese adults (Chen et al., 2020), it is possible that female adolescents are as efficient as male adolescents in concealing their facial expressions, corroborating the present results on suppression. Although suppressing emotions is generally preferred by individuals from a Chinese cultural background, females still express their emotions more freely than males (Zhao et al., 2014). Therefore, compared to their male counterparts, female teenagers tend to be more expressive and subsequently develop greater expressive abilities. This might explain the gender differences in expressive enhancement ability observed in the present study. However, caution should be taken when interpreting the effect of sex on overall EF scores because this effect had borderline significance (*p* = 0.065), and the effect sizes were relatively small (*d* = 0.13).

It is worth noting that, despite focusing on different dimensions of emotional intelligence, relevant research has also reported significant gender differences. A recent study found that adolescent boys showed higher levels of emotional intelligence in terms of self-emotion appraisal, use of emotions, and regulation of emotions, whereas adolescent girls presented higher levels of other-emotion appraisal (Costa et al., 2021). Notably, theoretical studies have suggested that the existence and magnitude of gender effects on emotional intelligence may be influenced by cultural and educational factors (Gebregergis et al., 2020). Given this context, cross-cultural research may be beneficial to test the above explanations concerning gender-related effects on emotional intelligence and its dimensions in adolescents, and gender-specific interventions could be designed to improve their EF.

### 4.3. Relationship between EF and depressive symptoms among Chinese adolescents

After controlling for age and sex, the results showed that higher EF and enhancement abilities were predictive of lower levels of depressive symptoms, while suppression ability was not. These findings accord with those of previous studies on the association between EF and depressive symptoms in older Chinese children and younger adolescents. Wang and Hawk (2020) also found that EF and enhancement abilities were significantly associated with lower levels of depressive symptoms but, in contrast to our study, they found that suppression ability was significantly associated with fewer depressive symptoms. Nevertheless, the results of this study may be attributed to the social norms surrounding the suppression and expression of emotions in China. Given that suppressing emotions is generally more contextually adaptive than expressing them in Chinese culture, Chinese people are more inclined to practice expressive suppression in their daily lives. Moreover, the Chinese may have a greater ability to suppress rather than express their emotions due to their collectivist culture. Therefore, the association between suppression ability and depressive symptoms may be less pronounced than the association between enhancement ability and depressive symptoms.

Similar to the present study, Rodin et al. (2016) reported that enhancement ability, but not suppression ability, was a significant predictor of lower levels of depressive symptoms and of PTSD symptoms in American combat veterans. Even though the participants in the study by Rodin et al. (2016) were from an individualistic rather than a collectivist culture, they were encouraged to suppress their emotional responses in work-related situations. Therefore, Rodin et al. (2016) results indirectly support our interpretation of our results concerning the closer association between enhancement ability and depressive symptoms. Further studies are needed to directly verify this interpretation in Chinese participants. Overall, the findings of this study suggest that EF and enhancement ability, but not suppression ability, are predictive of lower levels of depressive symptoms in Chinese adolescents.

Although previous studies have found that self-reported emotional intelligence is negatively related to depressive symptoms in adolescents (Fernández-Berrocal et al., 2006; Salguero et al., 2012; Resurrección et al., 2014; Gomez-Baya et al., 2017; Gardner and Lambert, 2019), this study is the first to investigate the potential relationships between a core facet of emotional intelligence (i.e., EF) and its dimensions and depressive symptoms in adolescents. The findings of the current study, namely, that enhancement and suppression abilities exhibit distinct influences on depressive symptoms, clarify the specific impact of enhancement and suppression abilities on adolescents’ depressive symptoms. They also suggest possible mechanisms underlying the influence of emotional intelligence on emotional well-being during adolescence. A recent empirical study reported that enhancement ability is associated with three specific TEI dimensions, namely, well-being, emotionality, and sociability, whereas suppression ability is associated with a fourth dimension, namely, self-control (Quattropani et al., 2022). It would be helpful to explore the role of EF as a mediator between emotional intelligence and psychological health. More importantly, specific interventions may be designed to boost adolescents’ emotional intelligence by cultivating EF and eventually decrease their depressive symptoms.

### 4.4. Limitations

This study had some limitations. First, it focused on a critical aspect of emotional intelligence, that is, the flexible utilization of enhancement and suppression. However, other aspects, such as having a flexible choice in terms of enhancement or suppression, are also important and require further investigation. Second, this study adopted a cross-sectional design to examine the development of EF and its association with depressive symptoms. However, this approach does not allow for an analysis of intra-individual changes in EF over time. It was also not possible to determine whether EF abilities were antecedents, concomitants, or consequences of the depressive symptoms. Therefore, future research employing longitudinal or experimental designs is necessary to address these deficiencies. Third, this study measured EF using the AFREE scale. Although the AFREE scale has been previously validated to assess EF abilities among Chinese teenagers, this self-report approach is susceptible to several methodological issues (e.g., social desirability). Therefore, further studies should use combined approaches (e.g., self-report scales, lab-based tasks, and realistic social interaction) to comprehensively evaluate the role of flexibility in regulating emotional expressions. Fourth, this study focused solely on the expressive regulation of general positive and negative emotions. According to previous empirical research on Chinese undergraduate students, greater suppression of sadness was associated with lower levels of depressive symptoms, while greater suppression of happiness was associated with higher levels of depressive symptoms (Zhou et al., 2016). Therefore, future research is needed to explore the specific ability or abilities involved when modulating emotional expression in individuals with depressive symptoms when confronted with discrete emotions (e.g., sadness, happiness, and fear). Finally, the participants of the present study were recruited exclusively from China. Since the distinct display rules for emotional expression in Chinese culture, which is considered a collectivist culture, it is necessary for future research to examine the cultural influence on the age and gender effects of EF, as well as its association with depressive symptoms in other cultural contexts (e.g., more individualistic culture).

## 5. Conclusion

To the best of our knowledge, this study is the first to assess EF in relation to age and gender in younger and older Chinese adolescents and the association of EF with depressive symptoms. The findings revealed that EF abilities remained stable throughout adolescence, that girls had greater expressive flexibility and enhancement abilities than boys, but not suppression ability, and that overall EF and enhancement abilities significantly predicted lower levels of depressive symptoms, while suppression ability did not. These findings deepen understanding of EF abilities and emotion regulation deficits among individuals with depressive symptoms.

## Data availability statement

The datasets presented in this article are not readily available because the datasets generated and/or analyzed during the current study are not publicly available due to time limitations. Requests to access the datasets should be directed to SZ, zhangshaohua0224@126.com.

## Ethics statement

The studies involving human participants were reviewed and approved by The Ethics Committee of East China Normal University (approval number: HR 121-2019). Written informed consent to participate in this study was provided by the participants’ legal guardian/next of kin.

## Author contributions

SZ: study design, data collection, analyses, interpretation, writing an original draft, and visualization. JL: clinical evaluations. BS: study design and clinical evaluations. YZ: study design and clinical evaluations. All authors contributed to the article and approved the submitted version.

## Funding

This work was financially supported by the National Natural Science Foundation of China (No. 31371043) to BS. The current study was also founded by the Shanghai Academy of Educational Sciences.

## Conflict of interest

The authors declare that the research was conducted in the absence of any commercial or financial relationships that could be construed as a potential conflict of interest.

## Publisher’s note

All claims expressed in this article are solely those of the authors and do not necessarily represent those of their affiliated organizations, or those of the publisher, the editors and the reviewers. Any product that may be evaluated in this article, or claim that may be made by its manufacturer, is not guaranteed or endorsed by the publisher.
